# Interaction Between Staphylococcal Biofilm and Bone: How Does the Presence of Biofilm Promote Prosthesis Loosening?

**DOI:** 10.3389/fmicb.2019.01602

**Published:** 2019-07-17

**Authors:** Jérôme Josse, Florent Valour, Yousef Maali, Alan Diot, Cécile Batailler, Tristan Ferry, Frédéric Laurent

**Affiliations:** ^1^CIRI - Centre International de Recherche en Infectiologie, Inserm U1111, CNRS UMR5308, ENS Lyon, Université Claude Bernard Lyon 1, Lyon, France; ^2^Université Claude Bernard Lyon 1, Université de Lyon, Lyon, France; ^3^Centre Interrégional de Référence des Infections Ostéo-articulaires Complexes (CRIOAc Lyon), Hospices Civils de Lyon, Lyon, France; ^4^Service de Chirurgie Orthopédique, Hôpital de la Croix-Rousse, Hospices Civils de Lyon, Lyon, France; ^5^Service de Maladies Infectieuses, Hôpital de la Croix-Rousse, Hospices Civils de Lyon, Lyon, France; ^6^Laboratoire de Bactériologie, Institut des Agents Infectieux, Hôpital de la Croix-Rousse, Hospices Civils de Lyon, Lyon, France

**Keywords:** prosthetic joint infection, prosthesis loosening, biofilm, *Staphylococcus*, polymorphonuclear neutrophil, osteoblast, osteoclast

## Abstract

With the aging of population, the number of indications for total joint replacement is continuously increasing. However, prosthesis loosening can happen and is related to two major mechanisms: (1) aseptic loosening due to prosthesis micromotion and/or corrosion and release of wear particles from the different components of the implanted material and (2) septic loosening due to chronic prosthetic joint infection (PJI). The “aseptic” character of prosthesis loosening has been challenged over the years, especially considering that bacteria can persist in biofilms and be overlooked during diagnosis. Histological studies on periprosthetic tissue samples reported that macrophages are the principle cells associated with aseptic loosening due to wear debris. They produce cytokines and favor an inflammatory environment that induces formation and activation of osteoclasts, leading to bone resorption and periprosthetic osteolysis. In PJIs, the presence of infiltrates of polymorphonuclear neutrophils is a major criterion for histological diagnosis. Neutrophils are colocalized with osteoclasts and zones of osteolysis. A similar inflammatory environment also develops, leading to bone resorption through osteoclasts. *Staphylococcus aureus*, *Staphylococcus epidermidis*, and *Staphylococcus lugdunensis* are the main staphylococci observed in PJIs. They share the common feature to form biofilm. For *S. aureus* and *S. epidermidis*, the interaction between biofilm and immunes cells (macrophages and polymorphonuclear neutrophils) differs regarding the species. Indeed, the composition of extracellular matrix of biofilm seems to impact the interaction with immune cells. Recent papers also reported the major role of myeloid-derived suppressor cells in biofilm-associated PJIs with *S. aureus*. These cells prevent lymphocyte infiltration and facilitate biofilm persistence. Moreover, the role of T lymphocytes is still unclear and potentially underestimates. In this review, after introducing the cellular mechanism of aseptic and septic loosening, we will focus on the interrelationships between staphylococcal biofilm, immune cells, and bone cells.

## Introduction

With the aging of population, the number of indications for total joint replacement is continuously increasing. The annual number of primary total hip and knee arthroplasties is projected to grow to 635,000 and 935,000 procedures by 2030, respectively (Sloan et al., 2018). However, prosthesis loosening can happen and is related to two major mechanisms: (1) aseptic loosening due to prosthesis micromotion and/or corrosion and release of wear particles from the different components of the implanted material and (2) septic loosening due to chronic prosthetic joint infection (PJI) (Morawietz et al., 2006). The “aseptic” character of prosthesis loosening has been challenged over the years, especially considering that bacteria can persist in biofilms and be overlooked during diagnosis (Nelson et al., 2005; Hoenders et al., 2008). In this review, we discuss the role of staphylococcal biofilm in prosthesis loosening, studying on *Staphylococcus aureus* (*S. aureus*) but also coagulase-negative staphylococci such as *Staphylococcus epidermidis* (*S. epidermidis*) and *Staphylococcus lugdunensis* (*S. lugdunensis*). After introducing the cellular mechanism of aseptic and septic loosening, we will focus on the interrelationships between staphylococcal biofilm, immune cells, and bone cells.

## Cellular Mechanisms Associated With Aseptic and Septic Loosening

### Osseointegration and Initial Foreign Body Response/Equilibrium

During the implantation of a foreign material, especially if this latter lacks of biocompatibility, an adverse innate host reaction, called foreign body response, can happen. Briefly, the several stages of the foreign body reaction include: (1) injury due to the implantation of material, (2) coating of the material with blood proteins and formation of temporary matrix, (3) an acute, and (4) a chronic inflammation (for an exhaustive description, see Anderson et al., 2008). If the chronic inflammation is not resolved, the host body finally shield off the material, enveloping it in a poorly vascularized fibrous layer (Albrektsson and Albrektsson, 1987). In bone tissue, a biocompatible material would normally be osseointegrated without the presence of any fibrous layer. The concept of osseointegration has been developed by a Swedish orthopedic surgeon called Per-Ingvar Brånemark that observed a direct bone formation in contact with metal implants when he implanted dental prostheses (Brånemark et al., 1969). However, a new paradigm has been recently proposed concerning osseointegration. Osseointegration would be a foreign body response where interfacial bone is formed to shield off the implant (Albrektsson et al., 2017). Technically, bone is a mineralized collagenous matrix with few cells inside, with similarities with the fibrous layer of the classical foreign body reaction. Concretely and regarding the size of an orthopedic prosthesis, osseointegration, and fibrous tissue capsule formation can potentially cohabit, with zones of direct interaction between bone and metal and zones where an interfacial fibrous layer is observed (Trindade et al., 2016).

### Periprosthetic Interface Membrane

During the retrieval of a prosthesis due to aseptic and septic loosening, a periprosthetic interface membrane, sometimes called synovium-like interface membrane (SLIM), can be observed at the interface between the implant and the bone (Morawietz et al., 2006). The origin of this membrane is not clear; it can potentially be related to the initial foreign body reaction or develops later after the implantation, in case of prosthesis micromotion or wear particle release.

This membrane is the location of cellular and enzymatic activities and production of pro-inflammatory and osteolytic mediators that lead to periprosthetic osteolysis (Hoenders et al., 2008). This membrane can also be observed in well-fixed implants, but is then considerably smaller (Goldring et al., 1983). Interface membrane is considered as the best histological sample for PJI diagnosis compared to bone biopsy, synovial fluid, or pseudocapsule (Bori et al., 2011). The cell composition of the interface membrane constitutes a major criterion to decipher if a prosthesis loosening is aseptic or septic (Morawietz et al., 2006). It should be noted that SLIM does not only referred to interface membrane in the Anglo-American literature but also to the synovial tissue and the regenerated synovial tissue. A detailed histological classification of SLIM exists, classifying the different patterns of periprosthetic tissue reactions (Krenn and Perino, 2017).

### Bone Cells and Osteolysis

**Figure 1:**
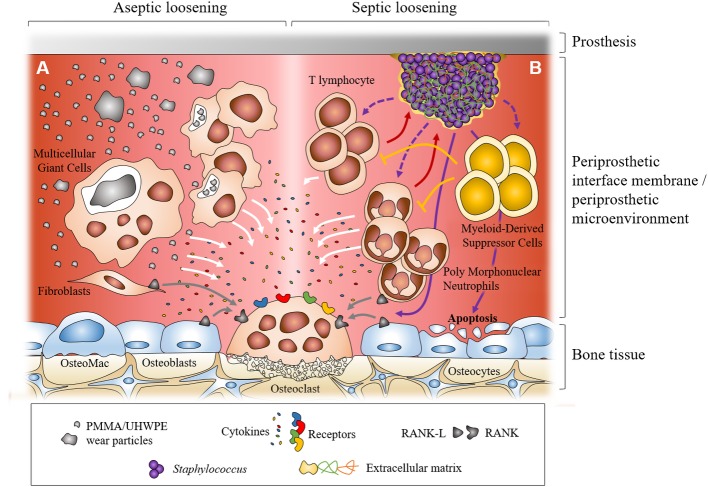
Dual representation of the major cellular mechanisms during aseptic and septic loosening at the interface between prosthesis and bone. In aseptic loosening, small wear debris from the prosthesis is phagocytosed by macrophages and bigger particles are phagocytosed by multicellular giant cells. Frustrated phagocytosis leads to the production of pro-inflammatory cytokines. Activated fibroblasts in the interfacial membrane can also produce RANKL **(A)**. In septic loosening, biofilm attached to the prosthesis can interact with polymorphonuclear neutrophils (PMNs) and T lymphocytes, which leads to the production of pro-inflammatory cytokines. Myeloid-derived suppressor cells (MDSCs) are recruited during biofilm-associated PJI. They can regulate immune response, notably inhibiting T lymphocyte proliferation. In PJI, recruited PMNs can express RANKL. Staphylococcal biofilm can also induce osteoblast apoptosis **(B)**. In both situations, pro-inflammatory cytokines and RANKL can trigger the formation and activation of osteoclasts which leads to bone resorption and prosthesis loosening.

Osteolysis, notably periprosthetic osteolysis, can potentially be related to a reduced bone formation and/or an enhanced bone resorption. Bone tissue is classically described as a mineralized matrix where three types of cells can be found: osteoblasts, osteocytes, and osteoclasts (Figure 1). Osteoblasts are the bone forming cells. They originate from mesenchymal stromal/stem cells that differentiate along their life. During their differentiation, cells from the osteoblastic lineage synthesize an organic matrix around them, mostly composed of type I collagen that is later mineralized (Blair et al., 2017). Mature osteoblasts finished as embedded in this mineralized matrix and differentiate into osteocytes (Abu-Amer and Tondravi, 1997). However, some osteoblasts remain on the surface of the new bone and differentiate into inactive bone-lining cells, forming an epithelial layer at the surface of the bone. The other osteoblasts undergo apoptosis (Abu-Amer and Tondravi, 1997; Blair et al., 2017).

Osteoclasts are responsible for the bone resorption. They rise from the fusion of monocytic precursors to form giant multinuclear cells. Bone resorption is operated by the release of H^+^ protons and proteases to resorb both inorganic and organic parts of the bone matrix (Adamopoulos and Mellins, 2015). Formation and activation of osteoclasts is controlled by the production of receptor activator of NF-κ B ligand (RANK-L) and osteoprotegerin (OPG). RANK-L interacts with its receptor RANK on monocyte/macrophage precursors to drive their differentiation toward osteoclasts whereas OPG is a soluble decoy receptor that targets RANK-L to limit osteoclastogenesis (Boyce and Xing, 2008).

In response to damage or biomechanical stimuli, osteocytes undergo apoptosis and release RANKL. It is the initial step that triggers the bone remodeling. Osteocytes also communicate with the endosteal lining cells that can form a canopy over the bone remodeling compartment (BRC). All the cells from the osteoblastic lineage can produce RANKL and OPG and modulate the osteoclastogenesis. Pro-inflammatory cytokines and chemokines can also favor the osteoclastogenesis, directly by interacting with the osteoclasts (Yokota et al., 2014) or indirectly by stimulating RANKL production by osteoblasts (Kwan Tat et al., 2004). Resident macrophages are also present in the bone microenvironment. These osteal macrophages, called OsteoMacs, are found in the canopy. They can express RANKL and are also responsible of the clearance of apoptotic osteoblasts by efferocytosis process (Batoon et al., 2017).

### Macrophages-Driven Osteolysis and Aseptic Loosening

Aseptic loosening can be due to two major factors. First, a lack of initial stability could lead to poor or absent osseointegration of the prosthesis. In this case, the loosening happens early after the implantation (Krismer et al., 1996). Second, the loosening can be related to the generation of wear debris coming from components of the prosthesis and/or the cement in case of cemented prosthesis. This process takes place slowly and develops chronically (Goodman, 2005). Wear debris can be generated either from joint replacement bearing surfaces and/or from the interfaces between the bone, bone cement (in case of cemented prosthesis), and the implant surface. Different types of wear debris can be produced: metal particles in case of metal-on-metal prosthesis, ultrahigh molecular weight polyethylene (UHWPE) particles in case of metal-on-polyethylene prosthesis, polymethylmethacrylate (PMMA) particles in case of cemented prosthesis (Pajarinen et al., 2014). These particles are released in the synovial fluid and can activate the cells present in the capsular neo-synovial membrane and periprosthetic interface membrane (SLIM). Histological studies performed on interface membrane retrieved during the implantation of a new prosthesis demonstrated that macrophages are the principal cells associated with prosthetic loosening due to wear particles (Goodman et al., 1998). Macrophages can be divided into two categories: (1) resident tissue macrophages (in our context, OsteoMacs) that contribute to tissue homeostasis and innate immune surveillance and (2) monocyte-derived macrophages that are recruited during damage and/or infection and orchestrate the innate and adaptative responses. Different subpopulations of resident macrophages are present in bone and bone marrow and contribute to bone homeostasis or hematopoiesis (Batoon et al., 2017).

In aseptic loosening due to wear debris, macrophages phagocyte the small wear debris which provoke the release of chemokines and pro-inflammatory cytokines that lead to further macrophage recruitment, osteoclast activation, and increased bone resorption (Pajarinen et al., 2014). Sabokar et al. also reported that human macrophages isolated from periprosthetic tissues can differentiate into osteoclast-like resorbing cells (Sabokbar et al., 1997). Macrophages can also fuse to form foreign body or multinuclear giant cells (FBGCs or MGCs), in order to phagocyte larger particles that cannot be taken in charge by macrophage alone. It has been shown that FBGCs have the capacity to dissolve the mineral phase of bone *in vivo*, in a way similar to osteoclasts. However, they are not able to digest the matrix fraction of bone, unlike osteoclasts (ten Harkel et al., 2015). In aseptic loosening context, it has also been reported that the differentiation of monocytes to mature osteoclasts can happen through the production of RANKL and TNF-α by activated fibroblasts which are present in the periprosthetic interface membrane (Sabokbar et al., 2005).

### Polymorphonuclear Neutrophils and Septic Loosening

Retrieved interface membranes after septic loosening reveal the presence of inflammatory infiltrates of polymorphonuclear neutrophils (PMNs), activated fibroblasts, and plasma cells (Morawietz et al., 2006). The presence of PMNs in periprosthetic tissue samples, such as interface membrane or synovial pseudocapsule, is an important criterion for the histological diagnosis of septic implant failure (Bori et al., 2018). According to the guidelines from the Musculoskeletal Infection Society (MSIS)/European Bone and Joint Society (EBJIS), the cutoff number of PMNs for the diagnostic of PJI is “*greater than five neutrophils per high-power field in five high-power fields observed from histologic analysis of periprosthetic tissue at ×400 magnification*” (Parvizi et al., 2011; Ochsner et al., 2014). However, low-grade infections, such as the ones due to *S. epidermidis* and other coagulase-negative staphylococci (CoNS), may be associated with a lower infiltration, and periprosthetic fractures may give false positive results on histological diagnosis (Bori et al., 2018). Concerning the role of PMNs in osteolysis, histological analysis of patients with osteomyelitis reported that the number of osteoclasts correlated with the abundance of infiltrated PMNs at the zones of bone resorption (Gaida et al., 2012). Chakravarti et al. observed that bacterial stimulation upregulated the expression of RANKL in PMNs, which stimulated bone resorption when co-cultured with osteoclasts (Chakravarti et al., 2009). Interestingly, it was also reported that PMNs can express RANK and be activated by RANKL. The expression of RANK by PMNs is upregulated in infected patients and could help for their recruitment to the infection site (Riegel et al., 2012). PMNs are also able to produce a large panel of cytokines and chemokines, potentially contributing to a pro-inflammatory environment and the activation of osteoclasts (Tecchio et al., 2014). They notably produce MRP-14, which was found upregulated in osteomyelitis and that stimulates the formation of osteoclasts (Dapunt et al., 2015). Finally, as observed for macrophages in aseptic loosening, the recruitment of PMNs in PJI leads to the generation of a pro-inflammatory environment that induces the formation and activation of osteoclasts (Gaida et al., 2012).

### T Lymphocytes

The analysis of periprosthetic interface membrane in aseptic or septic prosthesis loosening also revealed the presence of other types of cells such as T lymphocytes and plasma cells. (Morawietz et al., 2006). Dapunt et al. (2014) notably reported the detection of activated T lymphocytes in infected patients but not in patients with aseptic loosening. In their expanded classification of the types of interface membrane, Krenn and Perino describe the presence of “*scattered T lymphocytes and plasma cells*” in wear-induced SLIM and specify that “*PMN are often associated with plasma cells and small lymphocytic aggregates*” in infection-induced SLIM (Krenn and Perino, 2017).

## Staphylococcal Biofilm

### *Staphylococcus aureus* and the Other Staphylococci

*Staphylococcus* genus gathers at least 47 species and 23 sub-species. Staphylococci are generally classified as coagulase-positive, such as *S. aureus*, *S. argenteus*, or *S. pseudintermedius*, but most are coagulase-negative species such *S. epidermidis*, *S. lugdunensis*, or *S. capitis* (Becker et al., 2014). In chronic PJI, the most isolated pathogens are staphylococci, especially *S. aureus*, *S. epidermidis*, and also *S. lugdunensis* that has been highlighted in recent studies about PJI (Douiri et al., 2016; Lourtet-Hascoët et al., 2016; Triffault-Fillit et al., 2019). Globally, three types of physiopathological mechanisms, depending of the studied species, can be involved in staphylococcal chronic PJI: formation of small colony variants (SCV) (see the review from Proctor et al., 2014), bacterial internalization in osteoblasts (Josse et al., 2015), and formation of biofilm (Paharik and Horswill, 2016). For SCV formation, it has been observed for *S. aureus*, *S. epidermidis*, and *S. lugdunensis* (Proctor et al., 1994; von Eiff et al., 1999; Askar et al., 2018). SCV get a slow metabolism that makes them more tolerant to antibiotics. Concerning the ability to be internalized, it was reported for *S. aureus* in several *in vitro* experiments and some clinical histological approaches reported internalized *S. aureus* in patients with BJI (Bosse et al., 2005; Sendi et al., 2006; Josse et al., 2015). Internalization of *S. epidermidis* in osteoblasts is more controversial. In *in vitro* models, two papers reported an almost total lack of internalization (Valour et al., 2013; Campoccia et al., 2016), whereas a recent paper by Perez and Patel reported the ability of two clinical strains of *S. epidermidis* to penetrate osteoblasts (Perez and Patel, 2018). However, the level of internalization is similar in the three papers (around 100 intracellular staphylococci per 100,000 osteoblasts). So the real question is “does this *in vitro* result is clinically relevant?” This is currently difficult to say as no work has reported the presence of intracellular *S. epidermidis* in a clinical PJI/BJI sample yet. Interestingly, *S. pseudintermedius*, a species mostly found in dogs and incriminated in few human PJI/BJI cases (Darlow et al., 2017), can also be internalized in human osteoblasts *in vitro* (Maali et al., 2016). Concerning *S. lugdunensis*, two papers reported its inability to penetrate inside osteoblasts *in vitro* and there is no clinical report of internalized *S. lugdunensis* in bone (Campoccia et al., 2016; Maali et al., 2016).

A common feature shared by *S. aureus*, *S. epidermidis*, and *S. lugdunensis* is the ability to form biofilm. It was demonstrated in *in vitro* models (Buxton et al., 1987; Richards et al., 1991; Frank and Patel, 2007) and also in clinical PJI samples. The presence of biofilm of *S. aureus* has been observed directly from bone cement retrieved during a revision surgery and biofilm-like aggregates have been observed free in synovial fluid, supporting the role of biofilm in PJI (Stoodley et al., 2008; Dastgheyb et al., 2015).

### Biofilm Formation, Extracellular Matrix, and Toxin Production

Biofilm is defined as a bacterial community which is metabolically heterogeneous and embedded in a self-produced extracellular matrix (Costerton et al., 1999). The bacterial community inside biofilm is heterogeneous. It is composed of active bacteria but also contains bacteria with slow down metabolism (López et al., 2010). Due to this dormant state, these bacteria are more tolerant to antibiotic and can be related to “persisters” (Waters et al., 2016). This could partly explain that bacteria structured in biofilm are more tolerant to antibiotics than planktonic bacteria, directly impacting the outcome of PJI management (Stewart, 2015). The extracellular matrix produced by the bacteria is the other important part of the biofilm, mediating inter-bacterial adhesion. This matrix is composed of polysaccharides, proteins, and extracellular DNA (eDNA). Production of polysaccharide intercellular adhesin (PIA), also known as poly-N-acetyl-β-(1-6)-glucosamine (PNAG), is mediated by genes contained in the *icaADBC* gene locus (Mack et al., 1994). PIA participates to biofilm accumulation especially in *S. epidermidis* (Mack et al., 1996) but also in *S. aureus* (Cramton et al., 1999). However, PIA-negative strains of *S. epidermidis* are also able to form biofilm in a polysaccharide-independent manner. Accumulation-associated protein (aap) present at the surface of *S. epidermidis* is able to mediate cell-to-cell adhesion and biofilm formation, after being processed through staphylococcal proteases or host PMNs proteases (Rohde et al., 2005). PIA-independent biofilm accumulation and intercellular adhesion can also be relative to multifunctional cell surface proteins such as extracellular matrix binding protein (Embp) in *S. epidermidis* (Christner et al., 2010) or Fibronectin binding proteins A and B (FnBP A/B) in meticillin-resistant *S. aureus* (MRSA) (O’Neill et al., 2008). Biofilm matrix can also be composed of eDNA, resulting from the autolysis of bacteria releasing their genomic DNA (Rice et al., 2007; Christner et al., 2012). In *S. aureus* biofilm, DNA release is controlled by *cid* and *lrg* genes while staphylococcal thermonuclease can degrade the eDNA to promote biofilm dispersal (Mann et al., 2009). It has been suggested that eDNA acts as an electrostatic net, interconnecting bacterial cells through extracellular matrix proteins that have been released from bacterial cytoplasm (Foulston et al., 2014; Dengler et al., 2015). Recently, Sugimoto et al. reported that eDNA is a matrix component for all the strains of a MRSA collection whereas PIA was observed only for a small number of isolates (Sugimoto et al., 2018).

Phenol-soluble modulins (PSM), a specific type of staphylococcal cytotoxins, can also play a role in matrix composition. PSM are mostly known as agents that favor the dispersal of *S. aureus* biofilm but they also help to structure the biofilm and to control the biofilm expansion (Periasamy et al., 2012). Schwartz et al. reported that PSM can aggregate to form amyloid fibers that can help to stabilize *S. aureus* biofilm (Schwartz et al., 2012).

However, Zheng et al. recently demonstrated that PSM amyloid formation may not be of major relevance for biofilm formation, even if they support that PSM can attach to eDNA in biofilm matrix (Zheng et al., 2018). These findings are supported another study by Graf et al. that recently reported that the extracellular matrix of *S. aureus* biofilm contains a large amount of toxins that keep their cytotoxic activities but also play a role in the integrity of biofilm, interacting with anionic components such as eDNA in the acidic matrix environment (Graf et al., 2019). These results could potentially change our idea of biofilm, moving from a shelter to a “bunker” that allows staphylococci to harm by producing toxins in an environment that protects them from antibiotics and immune cells. Concerning *S. epidermidis*, PSM also play a role in biofilm structure *in vitro* and dispersal *in vitro* and *in vivo* (Wang et al., 2011). It was recently observed that no PSM from *a* can form amyloid fibers and that PSM do not seem to play a role in biofilm expansion (Le et al., 2019).

Host plasma proteins can also be integrated to biofilm matrix. Indeed, *S. aureus* can turn soluble fibrinogen into a fibrin shield thanks to its coagulase (Coa) or its von Willebrand factor-binding protein (vWbp), allowing an increased tolerance to antibiotics and to antimicrobial drugs (Zapotoczna et al., 2015). Regarding *S. lugdunensis*, which is less studied as it was more recently discovered, Frank and Patel showed its ability to form biofilm *in vitro*. They also reported that the biofilm extracellular matrix of *S. lugdunensis* is mostly made of proteins with a small quantity of PIA (Frank and Patel, 2007). Other reports have also described the role of the autolysin atlL in biofilm formation and the role of a competence gene, *comEB*, in DNA-dependent biofilm formation (Hussain et al., 2015; Rajendran et al., 2015).

## Interaction Between Biofilm and Host Cells

Staphylococci, especially *S. aureus*, have developed various strategies to subvert the host immune responses when growing as planktonic mode (Thammavongsa et al., 2015). In addition, biofilm formation can also be defined as a mean to protect from immune cells, in order to persist and develop chronic infections. Here, we focus on interaction between staphylococcal biofilm and immune cells that can be found in PJI (PMNs, macrophages, and myeloid-derived suppressor cells).

### Polymorphonuclear Neutrophils

PMNs are the first line of defense in PJI. Numerous infiltrates of PMNs are observed in infection-induced interface membrane (Morawietz et al., 2006). They can phagocyte planktonic bacteria previously opsonized or not by immunoglobulins IgG or complement component C3b. They can also release bactericidal components such as reactive oxygen species or enzymes (McGuinness et al., 2016). However, their ability to eliminate staphylococcal biofilm differs from one species to another. Indeed, PMNs can migrate toward and into *S. aureus* biofilm and clear it by phagocytosis. The biofilm clearance of *S. aureus* depends on the maturation state. Indeed, a mature biofilm seems more resistant to phagocytosis than a young one (Günther et al., 2009). Following phagocytosis of *S. aureus* biofilm, PMNs go apoptosis in order to prevent spilling of the bactericidal and cytotoxic entities (Guenther et al., 2009). Biofilm of *S. aureus* induces phagocytosis by PMNs but also degranulation of lactoferrin and elastase and DNA release. A partial destruction of biofilm has been observed *in vitro*, thereby supporting that biofilm structure does not completely protect staphylococci against the attack of PMNs (Meyle et al., 2010). Oxygen radical production by the PMNs also participates to the clearance of *S. aureus* biofilm and is dependent on opsonization by IgG (Stroh et al., 2011). Lei et al. (2019) recently reported that quorum sensing dysfunctionality favors resistance to PMNs in *S. aureus* biofilm infection. Indeed, accessory gene regulator (agr), the quorum sensing system of *S. aureus*, controls the production of mostly all the toxins (especially PSM) and enzymes and participate to the structuring of the biofilm. When *agr* gene or *psm* gene is deleted, the biofilm is more compact and the penetration of PMNs inside biofilm is more difficult. In opposite, biofilm formed by wild type strain is subject to PMNs phagocytosis (Lei et al., 2019).

Concerning the release of DNA by PMNs, also known as neutrophil extracellular trap (NET), a recent paper showed that the antimicrobial activity of released NETs are ineffective at clearing biofilm of *S. aureus* (Bhattacharya et al., 2018). At the opposite, NETs could potentiate the biofilm infections as eDNA was demonstrated to promote the biofilm formation (Dapunt et al., 2016a,b). Bhattacharya et al. also reported that leukocidins produced by *S. aureus* are required for NETosis (Bhattacharya et al., 2018). Interestingly, *S. epidermidis* appears more resistant to phagocytosis by PMNs and induce less apoptosis than *S. aureus* (Guenther et al., 2009). This phenomenon has been related to a difference in the composition of the extracellular biofilm matrix that may affect the motility of PMNs on biofilms and the ability to phagocyte. Indeed, production of PIA and Embp by *S. epidermidis* reduced biofilm phagocytosis by PMNs (Vuong et al., 2004; Christner et al., 2010). Opsonization by C3b and IgG has also been reported to be diminished for PIA-positive biofilm of *S. epidermidis* compared to PIA-negative ones (Kristian et al., 2008). Cerca et al. suggested that the high levels of PIA within the biofilm prevented a specific bacterial opsonization (Cerca et al., 2006). However, Meyle et al. reported that biofilms of *S. epidermidis*, similar to *S. aureus*, can activate PMNs leading to the release of cytotoxic and bactericidal components through an extracellular component, the bacterial heat shock protein GroEL (Meyle et al., 2012). Ferreirinha et al. observed in an *in vivo* mouse model that PIA-producing *S. epidermidis* enables a faster recruitment of PMNs and bacterial clearance compared to a PIA-defective isogenic mutant (Ferreirinha et al., 2016). These results support older experiments that reported an increased phagocytosis and an increased superoxide production by PMNs where they are challenged by a slime-producing strains of *S. epidermidis* compared to a slime non-producing strains (Heinzelmann et al., 1997). Finally, as it was observed for *S. aureus*, *S. epidermidis* biofilm (especially GroEL) is able to induce the formation of NET by PMNs and the release of MRP-14 (Dapunt et al., 2016a,b). So even if *S. epidermidis* in biofilm seems less impacted by PMNs, it is not totally protected from their activity and can activate the release of cytokines.

### Macrophages

Classically, planktonic staphylococci normally induce a proinflammatory microbicidal phenotype in macrophages. It implies the phagocytosis of bacteria, the production of bactericidal components and the release of pro-inflammatory cytokines such as IL-1β, IL-6, or TNF-ɑ (Flannagan et al., 2015). However, biofilm phenotype has been reported to protect *S. epidermidis* from phagocytosis and to limit the production of pro-inflammatory cytokines by macrophages, regardless of the morphotype (PIA, Embp, or Aap) (Shiau and Wu, 1998; Christner et al., 2010; Schommer et al., 2011). This inflammatory activation has been reported to be proportional to the level of dormant bacteria inside biofilm (Cerca et al., 2011). Spiliopoulou et al. reported that biofilm-associated bacteria can persist longer intracellularly after being phagocytosed by macrophages compared to planktonic staphylococci (Spiliopoulou et al., 2012a,b). Concerning *S. aureus* biofilms, *in vitro* studies demonstrated that macrophages can invade biofilms but display limited phagocytosis (Thurlow et al., 2011). A downregulation of the inducible nitric oxide synthase (iNOS), a major microbicidal mechanism, happens when macrophages are co-cultured with *S. aureus* biofilm whereas an increase of arginase-1 (Arg-1) expression, an enzyme that uses arginine to produce proline, a precursor for collagen, is observed. Indeed, when exposed to *S. aureus* biofilm, macrophages can shift from pro-inflammatory microbicidal phenotype (sometimes called M1) to an alternative phenotype, displaying anti-inflammatory and pro-fibrotic properties and limited phagocytosis (sometimes called M2) (Hanke et al., 2012). Using conditioned medium from *in vitro* biofilm culture, Scherr et al. reported that specific toxins, alpha-toxin (Hla) and leukocidin AB (LukAB), released by *S. aureus* biofilm act synergistically to inhibit macrophage phagocytosis and induce cytotoxicity, promoting macrophage dysfunction and thus facilitating *S. aureus* biofilm development (Scherr et al., 2015). Moreover, *S. aureus* biofilms release c-di-AMP, an important bacterial second messenger, *via* bacterial cell lysis to induce macrophage type I interferon production. This favors the intracellular survival of phagocyted *S. aureus* and promotes macrophage anti-inflammatory activity (Gries et al., 2016). In accordance with the previous findings, Alboslemy et al. recently reported that biofilm-conditioned medium can attenuates the activation of NF-κB in murine macrophages, a transcription factor involved in pro-inflammatory response, whereas it increases the expression of Kruppel-like factor (KLF2), a transcription regulator (Alboslemy et al., 2019). The authors suggest that secreted factors can hijack KLF2-dependent regulatory pathway to favor an anti-inflammatory responses in *S. aureus* biofilm-associated infections.

However, these previous findings have to be interpreted with precaution. Indeed, few macrophages are observed in histological samples from PJI (Morawietz et al., 2006). Even if the quantity of PMNs infiltrates is lower in low-grade infections, no clinical data concerning an increase of macrophages in periprosthetic tissues, with pro- or anti-inflammatory properties, have been reported during PJI.

### Myeloid-Derived Suppressor Cells

Myeloid-derived suppressor cells (MDSCs) are a heterogeneous population of immature monocytes and granulocytes with immunosuppressive properties, especially T lymphocyte inhibition. MDSCs can be divided into two groups: granulocytic or granulocytic MDSCs (G-MDSCs or PMN-MDSCs) and monocytic MDSCs (M-MDSCs), regarding their phenotypical and morphological similarities with PMNs or monocytes respectively (Veglia et al., 2018). In the recent years, MDSCs have been described as critical players in the regulation of inflammatory processes. In 2015, Tebartz et al. reported that *S. aureus* chronic infections are associated with immunosuppressive mechanisms. These latter are not driven by regulatory T lymphocytes but mostly by MDSCs (Tebartz et al., 2015). In a murine model of *S. aureus* biofilm infection, Heim et al. reported the presence of MDSCs surrounding biofilm. MDSCs are also able to attenuate macrophage pro-inflammatory activity and favor biofilm persistence (Heim et al., 2014). The recruitment of MDSCs is related to the release of IL-12 in infected tissues, whereas the anti-inflammatory properties of MDSCs are orchestrated by the release of IL-10 (Heim et al., 2015a,b). Interestingly, Peng et al. (2017) reported that the *in vitro* co-culture of bone marrow cells with *S. aureus* biofilm promotes the expansion of M-MDSCs but not G-MDSCs. Moreover, *S. aureus* biofilm is capable of stimulating the conversion of monocytic MDSCs into macrophages with anti-inflammatory properties *in vitro* and *in vivo* (Peng et al., 2017). However, MDSC infiltrates observed in PJI *in vivo* seems to be mostly granulocytic (Heim et al., 2018a). The granulocytic character of MDSCs has been confirmed in clinical samples from PJI patients. G-MDSCs could be used as infection markers as they were observed in samples associated with PJI but not to aseptic loosening (Heim et al., 2018b).

### T Lymphocytes in Staphylococcal Biofilm-Associated Prosthetic Joint Infection

Only few papers reported findings about the interaction between T lymphocytes and staphylococcal biofilm in PJI. However, lymphocyte infiltrates have been observed in interface membrane surrounding infected prosthesis (Morawietz et al., 2006). In 2011, Prabhakara et al. developed a chronic model of PJI in C57BL/6 mice, mimicking human biofilm-associated PJI as the infection was recalcitrant to clearance by the host immune response and antibiotics. They observed early Th1 and Th17 inflammatory responses whereas Th2 and Treg responses were downregulated in the same time. The authors suggest “*that staphylococcal biofilm infection resulted in the skewing of the host immune response toward proinflammatory Th1 and Th17 responses, which fail to clear the infection*” (Prabhakara et al., 2011a). Few months later, Prabhakara et al. published another study using a similar model in BALB/C mice. In this model, biofilm is naturally cleared and higher levels of IL-4 and IL-10 (Th2 cytokines) are observed as well as regulatory T lymphocytes. The authors suggested that Th2 response has a protective role against biofilm-associated infections and that inflammatory immune response is detrimental for the clearance of bacteria (Prabhakara et al., 2011b). In the same period, Leech et al. demonstrated a Janus-face role of IL-10 and T lymphocytes in systemic peritonitis infection or localized skin infection (Leech et al., 2017). In the localized infection, which could potentially related to biofilm, IL-10 production by MDSCs and macrophages inhibited the activation of T lymphocytes. This leads to the persistence of the infection, as observed in a murine PJI model by Heim et al. (2015a,b). These opposite results revealed that the role of T lymphocytes in biofilm-associated infections is still not clear and depend of the studied model or the site of infection. However, these last results have not been obtained in an experimental PJI model.

### Osteoclasts

Several studies reported that *S. aureus* or its components can favor bone resorption through direct interaction with the osteoclast cell lineage. Surface-associated material (SAM) from *S. aureus*, notably lipoproteins, was reported to stimulate osteoclast formation and bone resorption *in vitro*. Meghji et al. (1998) and Kim et al. (2013) have notably showed that RANKL does not play a major role in osteoclast formation in the presence of *S. aureus* SAM and that *S. aureus* SAM contains a soluble factor that promotes osteoclast formation by a RANKL-independent mechanism (Lau et al., 2006). It could potentially be the peptidoglycans from *S. aureus* which were reported to activate osteoclastogenesis through TLR 6/2 and NF-kB/NFAcT1 signaling pathway (Cao et al., 2017). A similar *in vitro* observation was done with *S. aureus* protein A (spA), provoking osteoclast differentiation and bone resorption (Ren et al., 2017). As it is known that spA activates the NF-kB signaling pathway in osteoblasts after linking to TNFR-1 (Claro et al., 2013), it seems logical to suggest that spA activation of bone resorption relies on its binding to TNFR-1 at the osteoclast surface. SpA was also reported to activate osteoclastogenesis through MAPK signaling (Wang et al., 2017). Trouillet-Assant et al. reported *in vitro* experiments that infection of osteoclast precursors by live planktonic *S. aureus* inhibits osteoclastogenesis but induces their differentiation into activated macrophages that actively secrete pro-inflammatory cytokines. These cytokines enhanced the bone resorption capacity of uninfected mature osteoclasts and promoted osteoclastogenesis of the uninfected precursors at the site of infection. The authors also reported that the infection of mature osteoclasts by *S. aureus* directly enhanced bone resorption by promoting cellular fusion (Trouillet-Assant et al., 2015). Toxins produced by *S. aureus* can also directly affect osteoclasts. Using recombinant toxins, Flammier et al. reported that osteoclasts displayed similar toxin susceptibility profiles compared to macrophages. Interestingly, toxic shock syndrome toxin 1 (TSST-1), mostly known for its role in menstrual toxic shock syndrome, was not cytotoxic but enhanced the bone resorption activity of osteoclasts (Flammier et al., 2016). However, all these results were obtained using *S. aureus* in its planktonic phenotype. Still, a direct effect of biofilm on bone was also observed and reported to directly resorb bone *in vitro* (Junka et al., 2017). As bone substrates used in this study were decellularized, the bone resorption cannot be related to osteoclastic activity. The authors suggest that the production of bacterial proteases can consequently be involved in a direct bone resorption. If this mechanism is confirmed, this ability to directly resorb bone tissue combined with the capacity to migrate into canaliculi and to form biofilm in osteocyte lacunae (de Mesy Bentley et al., 2017) could explain the ability of biofilm to induce periprosthetic osteolysis.

### Osteoblasts

Periprosthetic osteolysis could also be provoked by the impact of staphylococci on osteoblasts. Indeed, several studies have reported that planktonic *S. aureus* could interact with osteoblasts and inhibit bone formation by three major mechanisms: (1) decreasing osteoblast activity; (2) inducing osteoblast death; or (3) inducing RANKL production to enhance osteoclast activities. These different aspects are reviewed here (Josse et al., 2015). Focusing especially on interaction between biofilm and osteoblasts, Sanchez et al. have reported that biofilm-conditioned medium from clinical *S. aureus* isolates reduced osteoblast viability and increased apoptosis. Osteoblastic differentiation and bone mineralization were also significantly inhibited when osteoblasts were treated with biofilm supernatant. The authors also showed that the exposure of osteoblasts to biofilm-conditioned medium resulted in an upregulated expression of RANK-L and increase in the RANK-L/OPG ratio, potentially leading to the formation and activation of osteoclasts (Sanchez et al., 2013). Recently, Reffuveille et al. reported *in vitro* that soluble factors produced by osteoblasts directly influence *S. aureus* adhesion and could contribute to biofilm formation, suggesting an impact of the bone environment on biofilm formation (Reffuveille et al., 2018).

## Conclusion

Histological analysis of periprosthetic tissues from staphylococcal PJI, especially interface membrane, reported the presence of infiltrates of PMNs and lymphocytes. However, the infiltration of PMNs is limited in low-grade infections and mostly seen in acute infections. The presence of PMNs seems to be associated with the development of an inflammatory environment that activates bone resorption by osteoclasts, leading to periprosthetic osteolysis. Colocalization of PMNs and osteoclasts at bone resorption sites during PJI supports this hypothesis. The presence of G-MDSCs has also been reported in chronic PJI as a favorable factor for staphylococcal biofilm persistence. The co-existence of inflammatory PMNs leading to osteolysis and anti-inflammatory G-MDSCs leading to biofilm persistence seems conflicting to explain the mechanism of biofilm-associated periprosthetic osteolysis. Moreover, biofilm from *S. aureus* or *S. epidermidis* have different behaviors when interacting with PMNs. The chronology of events may be the key to explain the roles of PMNs and G-MDSCs in septic loosening. First, the interaction between staphylococcal biofilm and PMNs could bring to the development of inflammation. Then, G-MDSCs would be recruited in response to the inflammation, inducing an anti-inflammatory environment that favors the biofilm persistence. Finally, the recruited G-MDSCs would turn to PMNs that induce osteoclast activation and bone resorption. The ability for biofilm to modulate PMNs/G-MDSCs populations could be the key to explain the prosthetic loosening in PJI.

## Author Contributions

JJ, FV, YM, and AD prepared the draft of the paper. CB, TF, and FL revised the version of the text.

### Conflict of Interest Statement

The authors declare that the research was conducted in the absence of any commercial or financial relationships that could be construed as a potential conflict of interest.

## References

[ref1] Abu-Amer Y. Tondravi M. M. (1997). NF-kappaB and bone: the breaking point. Nat. Med. 3, 1189–1190. 10.1038/nm1197-1189, PMID: 9359684

[ref2] Adamopoulos I. E. Mellins E. D. (2015). Alternative pathways of osteoclastogenesis in inflammatory arthritis. Nat. Rev. Rheumatol. 11, 189–194. 10.1038/nrrheum.2014.198, PMID: 25422000 PMC4346500

[ref3] Alboslemy T. Yu B. Rogers T. Kim M.-H. (2019). *Staphylococcus aureus* biofilm-conditioned medium impairs macrophage-mediated antibiofilm immune response by upregulating KLF2 expression. Infect. Immun. 87:e00643-18. 10.1128/IAI.00643-18, PMID: 30692179 PMC6434135

[ref4] Albrektsson T. Albrektsson B. (1987). Osseointegration of bone implants. A review of an alternative mode of fixation. Acta Orthop. Scand. 58, 567–577. 3321881 10.3109/17453678709146401

[ref5] Albrektsson T. Chrcanovic B. Jacobsson M. Wennerberg A. (2017). Osseointegration of implants – a biological and clinical overview. JSM Dent. Surg. 2:1022.

[ref6] Anderson J. M. Rodriguez A. Chang D. T. (2008). Foreign body reaction to biomaterials. Semin. Immunol. 20, 86–100. 10.1016/j.smim.2007.11.004, PMID: 18162407 PMC2327202

[ref7] Askar M. Bloch B. Bayston R. (2018). Small-colony variant of *Staphylococcus lugdunensis* in prosthetic joint infection. Arthroplast. Today 4, 257–260. 10.1016/j.artd.2018.06.003, PMID: 30186900 PMC6123340

[ref8] Batoon L. Millard S. M. Raggatt L. J. Pettit A. R. (2017). Osteomacs and bone regeneration. Curr. Osteoporos. Rep. 15, 385–395. 10.1007/s11914-017-0384-x, PMID: 28647885

[ref556] Becker K. Heilmann C. Peters G. (2014). Coagulase-negative staphylococci. Clin. Microbiol. Rev. 27, 870–926. 10.1128/CMR.00109-13, PMID: 25278577 PMC4187637

[ref9] Bhattacharya M. Berends E. T. M. Chan R. Schwab E. Roy S. Sen C. K.. (2018). *Staphylococcus aureus* biofilms release leukocidins to elicit extracellular trap formation and evade neutrophil-mediated killing. Proc. Natl. Acad. Sci. USA 115, 7416–7421. 10.1073/pnas.1721949115, PMID: 29941565 PMC6048508

[ref10] Blair H. C. Larrouture Q. C. Li Y. Lin H. Beer-Stoltz D. Liu L.. (2017). Osteoblast differentiation and bone matrix formation *in vivo* and *in vitro*. Tissue Eng. B Rev. 23, 268–280. 10.1089/ten.teb.2016.0454, PMID: 27846781 PMC5467150

[ref11] Bori G. McNally M. A. Athanasou N. (2018). Histopathology in periprosthetic joint infection: when will the morphomolecular diagnosis be a reality? Biomed. Res. Int. 2018:1412701. 10.1155/2018/1412701, PMID: 29862251 PMC5971260

[ref12] Bori G. Muñoz-Mahamud E. Garcia S. Mallofre C. Gallart X. Bosch J.. (2011). Interface membrane is the best sample for histological study to diagnose prosthetic joint infection. Mod. Pathol. 24, 579–584. 10.1038/modpathol.2010.219, PMID: 21131917

[ref557] Bosse M. J. Gruber H. E. Ramp W. K. (2005). Internalization of bacteria by osteoblasts in a patient with recurrent, long-term osteomyelitis. A case report. J. Bone Joint Surg. Am. 87, 1343–1347. 10.2106/JBJS.D.02649, PMID: 15930546

[ref13] Boyce B. F. Xing L. (2008). Functions of RANKL/RANK/OPG in bone modeling and remodeling. Arch. Biochem. Biophys. 473, 139–146. 10.1016/j.abb.2008.03.018, PMID: 18395508 PMC2413418

[ref14] Brånemark P. I. Adell R. Breine U. Hansson B. O. Lindström J. Ohlsson A. (1969). Intra-osseous anchorage of dental prostheses. I. Experimental studies. Scand. J. Plast. Reconstr. Surg. 3, 81–100. 4924041 10.3109/02844316909036699

[ref15] Buxton T. B. Horner J. Hinton A. Rissing J. P. (1987). *In vivo* glycocalyx expression by *Staphylococcus aureus* phage type 52/52A/80 in *S. aureus* osteomyelitis. J. Infect. Dis. 156, 942–946. 10.1093/infdis/156.6.942, PMID: 3680993

[ref16] Campoccia D. Testoni F. Ravaioli S. Cangini I. Maso A. Speziale P.. (2016). Orthopedic implant infections: incompetence of *Staphylococcus epidermidis*, *Staphylococcus lugdunensis*, and *Enterococcus faecalis* to invade osteoblasts. J. Biomed. Mater. Res. A 104, 788–801. 10.1002/jbm.a.35564, PMID: 26378773

[ref17] Cao F. Zhou W. Liu G. Xia T. Liu M. Mi B.. (2017). *Staphylococcus aureus* peptidoglycan promotes osteoclastogenesis *via* TLR2-mediated activation of the NF-κB/NFATc1 signaling pathway. Am. J. Transl. Res. 9, 5022–5030. PMID: 29218100 PMC5714786

[ref18] Cerca F. Andrade F. França Â. Andrade E. B. Ribeiro A. Almeida A. A.. (2011). *Staphylococcus epidermidis* biofilms with higher proportions of dormant bacteria induce a lower activation of murine macrophages. J. Med. Microbiol. 60, 1717–1724. 10.1099/jmm.0.031922-0, PMID: 21799197 PMC10727147

[ref19] Cerca N. Jefferson K. K. Oliveira R. Pier G. B. Azeredo J. (2006). Comparative antibody-mediated phagocytosis of *Staphylococcus epidermidis* cells grown in a biofilm or in the planktonic state. Infect. Immun. 74, 4849–4855. 10.1128/IAI.00230-06, PMID: 16861673 PMC1539625

[ref20] Chakravarti A. Raquil M.-A. Tessier P. Poubelle P. E. (2009). Surface RANKL of toll-like receptor 4-stimulated human neutrophils activates osteoclastic bone resorption. Blood 114, 1633–1644. 10.1182/blood-2008-09-178301, PMID: 19546479

[ref21] Christner M. Franke G. C. Schommer N. N. Wendt U. Wegert K. Pehle P.. (2010). The giant extracellular matrix-binding protein of *Staphylococcus epidermidis* mediates biofilm accumulation and attachment to fibronectin. Mol. Microbiol. 75, 187–207. 10.1111/j.1365-2958.2009.06981.x, PMID: 19943904

[ref22] Christner M. Heinze C. Busch M. Franke G. Hentschke M. Bayard Dühring S.. (2012). sarA negatively regulates *Staphylococcus epidermidis* biofilm formation by modulating expression of 1MDa extracellular matrix binding protein and autolysis-dependent release of eDNA. Mol. Microbiol. 86, 394–410. 10.1111/j.1365-2958.2012.08203.x, PMID: 22957858

[ref23] Claro T. Widaa A. McDonnell C. Foster T. J. O’Brien F. J. Kerrigan S. W. (2013). *Staphylococcus aureus* protein A binding to osteoblast tumour necrosis factor receptor 1 results in activation of nuclear factor kappa B and release of interleukin-6 in bone infection. Microbiology 159, 147–154. 10.1099/mic.0.063016-0 23154968

[ref24] Costerton J. W. Stewart P. S. Greenberg E. P. (1999). Bacterial biofilms: a common cause of persistent infections. Science 284, 1318–1322. 10.1126/science.284.5418.1318, PMID: 10334980

[ref559] Cramton S. E. Gerke C. Schnell N. F. Nichols W. W. Götz F. (1999). The intercellular adhesion (ica) locus is present in Staphylococcus aureus and is required for biofilm formation. Infect. Immun. 67, 5427–5433. PMID: 10496925 10.1128/iai.67.10.5427-5433.1999 PMC96900

[ref25] Dapunt U. Gaida M. M. Meyle E. Prior B. Hänsch G. M. (2016a). Activation of phagocytic cells by *Staphylococcus epidermidis* biofilms: effects of extracellular matrix proteins and the bacterial stress protein GroEL on netosis and MRP-14 release. Pathog. Dis. 74:ftw035. 10.1093/femspd/ftw035 27109773 PMC5985485

[ref600] Dapunt U. Giese T. Maurer S. Stegmaier S. Prior B. Hänsch G. M.. (2015). Neutrophil‐derived MRP‐14 is up‐regulated in infectious osteomyelitis and stimulates osteoclast generation. J. Leukocyte Biology 98, 575–582. 10.1189/jlb.3VMA1014-482R, PMID: 25765681

[ref26] Dapunt U. Hänsch G. M. Arciola C. R. (2016b). Innate immune response in implant-associated infections: neutrophils against biofilms. Materials 9, pii: E387. 10.3390/ma9050387 28773509 PMC5503022

[ref555] Dapunt U. Maurer S. Giese T. Gaida M. M. Hänsch G. M. (2014). The Macrophage Inflammatory Proteins MIP1 (CCL3) and MIP2 (CXCL2) in Implant-Associated Osteomyelitis: Linking Inflammation to Bone Degradation. Mediators Inflamm. 2014, 1–10. 10.1155/2014/728619 PMC3984830 24795505

[ref27] Darlow C. A. Paidakakos N. Sikander M. Atkins B. (2017). A spinal infection with *Staphylococcus pseudintermedius*. BMJ Case Rep. 2017, pii: bcr-2017-221260. 10.1136/bcr-2017-221260, PMID: 28784907 PMC5624009

[ref28] Dastgheyb S. S. Hammoud S. Ketonis C. Liu A. Y. Fitzgerald K. Parvizi J.. (2015). staphylococcal persistence due to biofilm formation in synovial fluid containing prophylactic cefazolin. Antimicrob. Agents Chemother. 59, 2122–2128. 10.1128/AAC.04579-14, PMID: 25624333 PMC4356782

[ref29] de Mesy Bentley K. L. Trombetta R. Nishitani K. Bello-Irizarry S. N. Ninomiya M. Zhang L.. (2017). Evidence of *Staphylococcus aureus* deformation, proliferation, and migration in canaliculi of live cortical bone in murine models of osteomyelitis. J. Bone Miner. Res. 32, 985–990. 10.1002/jbmr.3055, PMID: 27933662 PMC5413415

[ref30] Dengler V. Foulston L. DeFrancesco A. S. Losick R. (2015). An electrostatic net model for the role of extracellular DNA in biofilm formation by *Staphylococcus aureus*. J. Bacteriol. 197, 3779–3787. 10.1128/JB.00726-15, PMID: 26416831 PMC4652055

[ref31] Douiri N. Hansmann Y. Lefebvre N. Riegel P. Martin M. Baldeyrou M.. (2016). *Staphylococcus lugdunensis*: a virulent pathogen causing bone and joint infections. Clin. Microbiol. Infect. 22, 747–748. 10.1016/j.cmi.2016.05.031, PMID: 27297318

[ref32] Ferreirinha P. Pérez-Cabezas B. Correia A. Miyazawa B. França A. Carvalhais V.. (2016). Poly-N-acetylglucosamine production by *Staphylococcus epidermidis* cells increases their in vivo proinflammatory effect. Infect. Immun. 84, 2933–2943. 10.1128/IAI.00290-16, PMID: 27481237 PMC5038083

[ref33] Flammier S. Rasigade J.-P. Badiou C. Henry T. Vandenesch F. Laurent F.. (2016). Human monocyte-derived osteoclasts are targeted by staphylococcal pore-forming toxins and superantigens. PLoS One 11:e0150693. 10.1371/journal.pone.0150693, PMID: 26934588 PMC4774977

[ref34] Flannagan R. S. Heit B. Heinrichs D. E. (2015). Antimicrobial mechanisms of macrophages and the immune evasion strategies of *Staphylococcus aureus*. Pathogens 4, 826–868. 10.3390/pathogens4040826, PMID: 26633519 PMC4693167

[ref35] Foulston L. Elsholz A. K. W. DeFrancesco A. S. Losick R. (2014). The extracellular matrix of *Staphylococcus aureus* biofilms comprises cytoplasmic proteins that associate with the cell surface in response to decreasing pH. MBio 5, e01667–e01614. 10.1128/mBio.01667-14, PMID: 25182325 PMC4173787

[ref36] Frank K. L. Patel R. (2007). Poly-N-acetylglucosamine is not a major component of the extracellular matrix in biofilms formed by icaADBC-positive *Staphylococcus lugdunensis* isolates. Infect. Immun. 75, 4728–4742. 10.1128/IAI.00640-07, PMID: 17635864 PMC2044555

[ref551] Gaida M. M. Mayer B. Stegmaier S. Schirmacher P. Wagner C. Hänsch G. M. (2012). Polymorphonuclear neutrophils in osteomyelitis: link to osteoclast generation and bone resorption. Eur. J. Inflamm. 413–426. 10.1177/1721727X1201000317, PMID: 6304106

[ref37] Goldring S. R. Schiller A. L. Roelke M. Rourke C. M. O’Neil D. A. Harris W. H. (1983). The synovial-like membrane at the bone-cement interface in loose total hip replacements and its proposed role in bone lysis. J. Bone Joint Surg. Am. 65, 575–584. 10.2106/00004623-198365050-00001, PMID: 6304106

[ref38] Goodman S. (2005). Wear particulate and osteolysis. Orthop. Clin. North Am. 36, 41–48, vi. 10.1016/j.ocl.2004.06.015 15542121

[ref39] Goodman S. B. Lind M. Song Y. Smith R. L. (1998). *In vitro*, *in vivo*, and tissue retrieval studies on particulate debris. Clin. Orthop. Relat. Res. 25–34. PMID: 9678030

[ref40] Graf A. C. Leonard A. Schäuble M. Rieckmann L. M. Hoyer J. Maaß S.. (2019). Virulence factors produced by *Staphylococcus aureus* biofilms have a moonlighting function contributing to biofilm integrity. Mol. Cell. Proteomics 18, 1036–1053. 10.1074/mcp.RA118.001120, PMID: 30850421 PMC6553939

[ref41] Gries C. M. Bruger E. L. Moormeier D. E. Scherr T. D. Waters C. M. Kielian T. (2016). Cyclic di-AMP released from *Staphylococcus aureus* biofilm induces a macrophage type I interferon response. Infect. Immun. 84, 3564–3574. 10.1128/IAI.00447-16, PMID: 27736778 PMC5116733

[ref42] Guenther F. Stroh P. Wagner C. Obst U. Hänsch G. M. (2009). Phagocytosis of staphylococci biofilms by polymorphonuclear neutrophils: *S. aureus* and *S. epidermidis* differ with regard to their susceptibility towards the host defense. Int. J. Artif. Organs 32, 565–573. 10.1177/039139880903200905 19856266

[ref43] Günther F. Wabnitz G. H. Stroh P. Prior B. Obst U. Samstag Y.. (2009). Host defence against *Staphylococcus aureus* biofilms infection: phagocytosis of biofilms by polymorphonuclear neutrophils (PMN). Mol. Immunol. 46, 1805–1813. 10.1016/j.molimm.2009.01.020, PMID: 19261332

[ref44] Hanke M. L. Angle A. Kielian T. (2012). MyD88-dependent signaling influences fibrosis and alternative macrophage activation during *Staphylococcus aureus* biofilm infection. PLoS One 7:e42476. 10.1371/journal.pone.0042476, PMID: 22879997 PMC3411753

[ref46] Heim C. E. Vidlak D. Kielian T. (2015a). Interleukin-10 production by myeloid-derived suppressor cells contributes to bacterial persistence during *Staphylococcus aureus* orthopedic biofilm infection. J. Leukoc. Biol. 98, 1003–1013. 10.1189/jlb.4VMA0315-125RR 26232453 PMC4661040

[ref47] Heim C. E. Vidlak D. Odvody J. Hartman C. W. Garvin K. L. Kielian T. (2018a). Human prosthetic joint infections are associated with myeloid-derived suppressor cells (MDSCs): implications for infection persistence. J. Orthop. Res. 36, 1605–1613. 10.1002/jor.23806 29139571 PMC5953848

[ref48] Heim C. E. Vidlak D. Scherr T. D. Hartman C. W. Garvin K. L. Kielian T. (2015b). IL-12 promotes myeloid-derived suppressor cell recruitment and bacterial persistence during *Staphylococcus aureus* orthopedic implant infection. J. Immunol. 194, 3861–3872. 10.4049/jimmunol.1402689 25762781 PMC4390492

[ref49] Heim C. E. Vidlak D. Scherr T. D. Kozel J. A. Holzapfel M. Muirhead D. E.. (2014). Myeloid-derived suppressor cells contribute to *Staphylococcus aureus* orthopedic biofilm infection. J. Immunol. 192, 3778–3792. 10.4049/jimmunol.1303408, PMID: 24646737 PMC4004612

[ref50] Heim C. E. West S. C. Ali H. Kielian T. (2018b). Heterogeneity of Ly6G+ Ly6C+ myeloid-derived suppressor cell infiltrates during *Staphylococcus aureus* biofilm infection. Infect. Immun. 86, pii: e00684-18. 10.1128/IAI.00684-18 PMC6246895 30249747

[ref51] Heinzelmann M. Herzig D. O. Swain B. Mercer-Jones M. A. Bergamini T. M. Polk H. C. (1997). Phagocytosis and oxidative-burst response of planktonic *Staphylococcus epidermidis* RP62A and its non-slime-producing variant in human neutrophils. Clin. Diagn. Lab. Immunol. 4, 705–710. PMID: 9384293 10.1128/cdli.4.6.705-710.1997 PMC170644

[ref52] Hoenders C. S. M. Harmsen M. C. van Luyn M. J. A. (2008). The local inflammatory environment and microorganisms in “aseptic” loosening of hip prostheses. J. Biomed. Mater. Res. B Appl. Biomater. 86, 291–301. 10.1002/jbm.b.30992, PMID: 18098200

[ref53] Hussain M. Steinbacher T. Peters G. Heilmann C. Becker K. (2015). The adhesive properties of the *Staphylococcus lugdunensis* multifunctional autolysin AtlL and its role in biofilm formation and internalization. Int. J. Med. Microbiol. 305, 129–139. 10.1016/j.ijmm.2014.11.010, PMID: 25515664

[ref54] Josse J. Velard F. Gangloff S. C. (2015). *Staphylococcus aureus* vs. osteoblast: relationship and consequences in osteomyelitis. Front. Cell. Infect. Microbiol. 5:85. 10.3389/fcimb.2015.00085, PMID: 26636047 PMC4660271

[ref55] Junka A. Szymczyk P. Ziółkowski G. Karuga-Kuzniewska E. Smutnicka D. Bil-Lula I.. (2017). Bad to the bone: on *in vitro* and *ex vivo* microbial biofilm ability to directly destroy colonized bone surfaces without participation of host immunity or osteoclastogenesis. PLoS One 12:e0169565. 10.1371/journal.pone.0169565, PMID: 28076372 PMC5226730

[ref56] Kim J. Yang J. Park O.-J. Kang S.-S. Kim W.-S. Kurokawa K.. (2013). Lipoproteins are an important bacterial component responsible for bone destruction through the induction of osteoclast differentiation and activation. J. Bone Miner. Res. 28, 2381–2391. 10.1002/jbmr.1973, PMID: 23633269

[ref57] Krenn V. Perino G. (2017). Histological diagnosis of implant-associated pathologies. Berlin/Heidelberg: Springer-Verlag. Available at: https://www.springer.com/gp/book/9783662542033 (Accessed May 9, 2019).

[ref58] Krismer M. Stöckl B. Fischer M. Bauer R. Mayrhofer P. Ogon M. (1996). Early migration predicts late aseptic failure of hip sockets. J. Bone Joint Surg. Br. 78, 422–426. PMID: 8636179

[ref59] Kristian S. A. Birkenstock T. A. Sauder U. Mack D. Götz F. Landmann R. (2008). Biofilm formation induces C3a release and protects *Staphylococcus epidermidis* from IgG and complement deposition and from neutrophil-dependent killing. J. Infect. Dis. 197, 1028–1035. 10.1086/528992, PMID: 18419540

[ref60] Kwan Tat S. Padrines M. Théoleyre S. Heymann D. Fortun Y. (2004). IL-6, RANKL, TNF-alpha/IL-1: interrelations in bone resorption pathophysiology. Cytokine Growth Factor Rev. 15, 49–60. 10.1016/j.cytogfr.2003.10.005, PMID: 14746813

[ref61] Lau Y. S. Wang W. Sabokbar A. Simpson H. Nair S. Henderson B. (2006). *Staphylococcus aureus* capsular material promotes osteoclast formation. Injury 37(Suppl. 2), S41–S48. 10.1016/j.injury.2006.04.008 16651071

[ref62] Le K. Y. Villaruz A. E. Zheng Y. He L. Fisher E. L. Nguyen T. H.. (2019). Role of phenol-soluble modulins in *Staphylococcus epidermidis* biofilm formation and infection of indwelling medical devices. J. Mol. Biol. pii: S0022-2836(19)30180-9. 10.1016/j.jmb.2019.03.030, PMID: 30954574 PMC10999989

[ref63] Leech J. M. Lacey K. A. Mulcahy M. E. Medina E. McLoughlin R. M. (2017). IL-10 plays opposing roles during *Staphylococcus aureus* systemic and localized infections. J. Immunol. 198, 2352–2365. 10.4049/jimmunol.1601018, PMID: 28167629 PMC5337812

[ref560] Lei H. Le K. Y. Khan B. A. Nguyen T. H. Hunt R. L. Bae J. S.. (2019). Resistance to leukocytes ties benefits of quorum sensing dysfunctionality to biofilm infection. Nat. Microbiol. 4, 1114–1119. 10.1038/s41564-019-0413-x, PMID: 30936487 PMC6588452

[ref64] López D. Vlamakis H. Kolter R. (2010). Biofilms. Cold Spring Harb. Perspect. Biol. 2:a000398. 10.1101/cshperspect.a000398, PMID: 20519345 PMC2890205

[ref65] Lourtet-Hascoët J. Bicart-See A. Félicé M. P. Giordano G. Bonnet E. (2016). *Staphylococcus lugdunensis*, a serious pathogen in periprosthetic joint infections: comparison to *Staphylococcus aureus* and *Staphylococcus epidermidis*. Int. J. Infect. Dis. 51, 56–61. 10.1016/j.ijid.2016.08.007, PMID: 27609028

[ref66] Maali Y. Martins-Simões P. Valour F. Bouvard D. Rasigade J.-P. Bes M.. (2016). Pathophysiological mechanisms of staphylococcus non-aureus bone and joint infection: interspecies homogeneity and specific behavior of *S. pseudintermedius*. Front. Microbiol. 7:1063. 10.3389/fmicb.2016.01063, PMID: 27462303 PMC4940379

[ref67] Mack D. Fischer W. Krokotsch A. Leopold K. Hartmann R. Egge H.. (1996). The intercellular adhesin involved in biofilm accumulation of *Staphylococcus epidermidis* is a linear beta-1,6-linked glucosaminoglycan: purification and structural analysis. J. Bacteriol. 178, 175–183. 10.1128/jb.178.1.175-183.1996, PMID: 8550413 PMC177636

[ref68] Mack D. Nedelmann M. Krokotsch A. Schwarzkopf A. Heesemann J. Laufs R. (1994). Characterization of transposon mutants of biofilm-producing *Staphylococcus epidermidis* impaired in the accumulative phase of biofilm production: genetic identification of a hexosamine-containing polysaccharide intercellular adhesin. Infect. Immun. 62, 3244–3253. PMID: 8039894 10.1128/iai.62.8.3244-3253.1994 PMC302952

[ref69] Mann E. E. Rice K. C. Boles B. R. Endres J. L. Ranjit D. Chandramohan L.. (2009). Modulation of eDNA release and degradation affects *Staphylococcus aureus* biofilm maturation. PLoS One 4:e5822. 10.1371/journal.pone.0005822, PMID: 19513119 PMC2688759

[ref70] McGuinness W. A. Kobayashi S. D. DeLeo F. R. (2016). Evasion of neutrophil killing by *Staphylococcus aureus*. Pathogens 5, pii: E32. 10.3390/pathogens5010032, PMID: 26999220 PMC4810153

[ref71] Meghji S. Crean S. J. Hill P. A. Sheikh M. Nair S. P. Heron K.. (1998). Surface-associated protein from *Staphylococcus aureus* stimulates osteoclastogenesis: possible role in *S. aureus*-induced bone pathology. Br. J. Rheumatol. 37, 1095–1101. 10.1093/rheumatology/37.10.1095, PMID: 9825749

[ref72] Meyle E. Brenner-Weiss G. Obst U. Prior B. Hänsch G. M. (2012). Immune defense against *S. epidermidis* biofilms: components of the extracellular polymeric substance activate distinct bactericidal mechanisms of phagocytic cells. Int. J. Artif. Organs 35, 700–712. 10.5301/ijao.5000151 23065886

[ref73] Meyle E. Stroh P. Günther F. Hoppy-Tichy T. Wagner C. Hänsch G. M. (2010). Destruction of bacterial biofilms by polymorphonuclear neutrophils: relative contribution of phagocytosis, DNA release, and degranulation. Int. J. Artif. Organs 33, 608–620. 10.1177/039139881003300906 20890882

[ref74] Morawietz L. Classen R.-A. Schröder J. H. Dynybil C. Perka C. Skwara A.. (2006). Proposal for a histopathological consensus classification of the periprosthetic interface membrane. J. Clin. Pathol. 59, 591–597. 10.1136/jcp.2005.027458, PMID: 16731601 PMC1860400

[ref75] Nelson C. L. McLaren A. C. McLaren S. G. Johnson J. W. Smeltzer M. S. (2005). Is aseptic loosening truly aseptic? Clin. Orthop. Relat. Res. 25–30. 10.1097/01.blo.0000175715.68624.3d, PMID: 16056022

[ref76] O’Neill E. Pozzi C. Houston P. Humphreys H. Robinson D. A. Loughman A.. (2008). A novel *Staphylococcus aureus* biofilm phenotype mediated by the fibronectin-binding proteins, FnBPA and FnBPB. J. Bacteriol. 190, 3835–3850. 10.1128/JB.00167-08, PMID: 18375547 PMC2395027

[ref77] Ochsner P. E. Borens O. Bodler P.-M. Schweizerische Gesellschaft für Orthopädie und Traumatologie (2014). Infections of the musculoskeletal system: Basic principles, prevention, diagnosis and treatment. Grandvaux Swiss orthopaedics in-house-publisher.

[ref78] Paharik A. E. Horswill A. R. (2016). The staphylococcal biofilm: adhesins, regulation, and host response. Microbiol. Spectr. 4:VMBF-0022-201. 10.1128/microbiolspec.VMBF-0022-2015, PMID: 27227309 PMC4887152

[ref79] Pajarinen J. Jamsen E. Konttinen Y. T. Goodman S. B. (2014). Innate immune reactions in septic and aseptic osteolysis around hip implants. J. Long-Term Eff. Med. Implants 24, 283–296. 10.1615/JLongTermEffMedImplants.2014010564, PMID: 25747031 PMC4366426

[ref550] Parvizi J. Zmistowski B. Berbari E. F. Bauer T. W. Springer B. D. Della Valle C. J.. (2011). New definition for periprosthetic joint infection: from the workgroup of the musculoskeletal infection society. Clin. Orthop. Relat. Res. 469, 2992–2994. 10.1007/s11999-011-2102-9, PMID: 21938532 PMC3183178

[ref570] Peng K.-T. Hsieh C.-C. Huang T.-Y. Chen P.-C. Shih H.-N. Lee M. S. (2017). Staphylococcus aureus biofilm elicits the expansion, activation and polarization of myeloid-derived suppressor cells *in vivo* and *in vitro*. PloS One 12:e0183271. 10.1371/journal.pone.0183271 28813499 PMC5559065

[ref80] Perez K. Patel R. (2018). Survival of *Staphylococcus epidermidis* in fibroblasts and osteoblasts. Infect. Immun. 86, pii: e00237-18. 10.1128/IAI.00237-18, PMID: 30061380 PMC6204734

[ref81] Periasamy S. Joo H.-S. Duong A. C. Bach T.-H. L. Tan V. Y. Chatterjee S. S. (2012). How *Staphylococcus aureus* biofilms develop their characteristic structure. Proc. Natl. Acad. Sci. USA 109, 1281–1286. 10.1073/pnas.1115006109 22232686 PMC3268330

[ref82] Prabhakara R. Harro J. M. Leid J. G. Harris M. Shirtliff M. E. (2011a). Murine immune response to a chronic *Staphylococcus aureus* biofilm infection. Infect. Immun. 79, 1789–1796. 10.1128/IAI.01386-10 21282411 PMC3067568

[ref83] Prabhakara R. Harro J. M. Leid J. G. Keegan A. D. Prior M. L. Shirtliff M. E. (2011b). Suppression of the inflammatory immune response prevents the development of chronic biofilm infection due to methicillin-resistant *Staphylococcus aureus*. Infect. Immun. 79, 5010–5018. 10.1128/IAI.05571-11 21947772 PMC3232664

[ref84] Proctor R. A. Balwit J. M. Vesga O. (1994). Variant subpopulations of *Staphylococcus aureus* as cause of persistent and recurrent infections. Infect. Agents Dis. 3, 302–312. PMID: 7889317

[ref85] Proctor R. A. Kriegeskorte A. Kahl B. C. Becker K. Löffler B. Peters G. (2014). *Staphylococcus aureus* small colony variants (SCVs): a road map for the metabolic pathways involved in persistent infections. Front. Cell. Infect. Microbiol. 4:99. 10.3389/fcimb.2014.00099, PMID: 25120957 PMC4112797

[ref86] Rajendran N. B. Eikmeier J. Becker K. Hussain M. Peters G. Heilmann C. (2015). Important contribution of the novel locus comEB to extracellular DNA-dependent *Staphylococcus lugdunensis* biofilm formation. Infect. Immun. 83, 4682–4692. 10.1128/IAI.00775-15, PMID: 26416910 PMC4645410

[ref87] Reffuveille F. Josse J. Velard F. Lamret F. Varin-Simon J. Dubus M.. (2018). Bone environment influences irreversible adhesion of a methicillin-susceptible *Staphylococcus aureus* strain. Front. Microbiol. 9:2865. 10.3389/fmicb.2018.02865, PMID: 30538688 PMC6277558

[ref88] Ren L.-R. Wang H. He X.-Q. Song M.-G. Chen X.-Q. Xu Y.-Q. (2017). *Staphylococcus aureus* protein a induces osteoclastogenesis via the NF-κB signaling pathway. Mol. Med. Rep. 16, 6020–6028. 10.3892/mmr.2017.7316, PMID: 28849198 PMC5865801

[ref89] Rice K. C. Mann E. E. Endres J. L. Weiss E. C. Cassat J. E. Smeltzer M. S. (2007). The cidA murein hydrolase regulator contributes to DNA release and biofilm development in *Staphylococcus aureus*. Proc. Natl. Acad. Sci. USA 104, 8113–8118. 10.1073/pnas.0610226104 17452642 PMC1876580

[ref90] Richards G. K. Gagnon R. F. Prentis J. (1991). Comparative rates of antibiotic action against *Staphylococcus epidermidis* biofilms. ASAIO Trans. 37, M160–M162. PMID: 1751091

[ref552] Riegel A. Maurer T. Prior B. Stegmaier S. Heppert V. Wagner C. (2012). Human polymorphonuclear neutrophils express RANK and are activated by its ligand, RANKL. Eur. J. Immunol. 42, 975–981. 10.1002/eji.201141786 22531921

[ref91] Rohde H. Burdelski C. Bartscht K. Hussain M. Buck F. Horstkotte M. A.. (2005). Induction of *Staphylococcus epidermidis* biofilm formation via proteolytic processing of the accumulation-associated protein by staphylococcal and host proteases. Mol. Microbiol. 55, 1883–1895. 10.1111/j.1365-2958.2005.04515.x, PMID: 15752207

[ref92] Sabokbar A. Fujikawa Y. Neale S. Murray D. W. Athanasou N. A. (1997). Human arthroplasty derived macrophages differentiate into osteoclastic bone resorbing cells. Ann. Rheum. Dis. 56, 414–420. 10.1136/ard.56.7.414 9486003 PMC1752416

[ref93] Sabokbar A. Itonaga I. Sun S. G. Kudo O. Athanasou N. A. (2005). Arthroplasty membrane-derived fibroblasts directly induce osteoclast formation and osteolysis in aseptic loosening. J. Orthop. Res. 23, 511–519. 10.1016/j.orthres.2004.10.006, PMID: 15885469

[ref94] Sanchez C. J. Ward C. L. Romano D. R. Hurtgen B. J. Hardy S. K. Woodbury R. L.. (2013). *Staphylococcus aureus* biofilms decrease osteoblast viability, inhibits osteogenic differentiation, and increases bone resorption in vitro. BMC Musculoskelet. Disord. 14:187. 10.1186/1471-2474-14-187, PMID: 23767824 PMC3691632

[ref95] Scherr T. D. Hanke M. L. Huang O. James D. B. A. Horswill A. R. Bayles K. W.. (2015). *Staphylococcus aureus* biofilms induce macrophage dysfunction through leukocidin AB and alpha-toxin. MBio 6, pii: e01021-15. 10.1128/mBio.01021-15, PMID: 26307164 PMC4550693

[ref96] Schommer N. N. Christner M. Hentschke M. Ruckdeschel K. Aepfelbacher M. Rohde H. (2011). *Staphylococcus epidermidis* uses distinct mechanisms of biofilm formation to interfere with phagocytosis and activation of mouse macrophage-like cells 774A.1. Infect. Immun. 79, 2267–2276. 10.1128/IAI.01142-10, PMID: 21402760 PMC3125858

[ref97] Schwartz K. Syed A. K. Stephenson R. E. Rickard A. H. Boles B. R. (2012). Functional amyloids composed of phenol soluble modulins stabilize *Staphylococcus aureus* biofilms. PLoS Pathog. 8:e1002744. 10.1371/journal.ppat.1002744, PMID: 22685403 PMC3369951

[ref558] Sendi P. Rohrbach M. Graber P. Frei R. Ochsner P. E. Zimmerli W. (2006). Staphylococcus aureus Small Colony Variants in Prosthetic Joint Infection. Clin. Infect. Dis. 43, 961–967. 10.1086/507633, PMID: 16983605

[ref98] Shiau A. L. Wu C. L. (1998). The inhibitory effect of *Staphylococcus epidermidis* slime on the phagocytosis of murine peritoneal macrophages is interferon-independent. Microbiol. Immunol. 42, 33–40. 10.1111/j.1348-0421.1998.tb01966.x, PMID: 9525777

[ref99] Sloan M. Premkumar A. Sheth N. P. (2018). Projected volume of primary total joint arthroplasty in the U.S., 2014 to 2030. J. Bone Joint Surg. Am. 100, 1455–1460. 10.2106/JBJS.17.01617, PMID: 30180053

[ref100] Spiliopoulou A. I. Kolonitsiou F. Krevvata M. I. Leontsinidis M. Wilkinson T. S. Mack D. (2012a). Bacterial adhesion, intracellular survival and cytokine induction upon stimulation of mononuclear cells with planktonic or biofilm phase *Staphylococcus epidermidis*. FEMS Microbiol. Lett. 330, 56–65. 10.1111/j.1574-6968.2012.02533.x 22360699

[ref101] Spiliopoulou A. I. Krevvata M. I. Kolonitsiou F. Harris L. G. Wilkinson T. S. Davies A. P. (2012b). An extracellular *Staphylococcus epidermidis* polysaccharide: relation to polysaccharide intercellular adhesin and its implication in phagocytosis. BMC Microbiol. 12:76. 10.1186/1471-2180-12-76 22594478 PMC3431232

[ref102] Stewart P. S. (2015). Antimicrobial tolerance in biofilms. Microbiol. Spectr. 3. 10.1128/microbiolspec.MB-0010-2014, PMID: 26185072 PMC4507308

[ref103] Stoodley P. Nistico L. Johnson S. Lasko L.-A. Baratz M. Gahlot V. (2008). Direct demonstration of viable *Staphylococcus aureus* biofilms in an infected total joint arthroplasty. A case report. J. Bone Joint Surg. Am. 90, 1751–1758. 10.2106/JBJS.G.00838 18676908 PMC2729478

[ref104] Stroh P. Günther F. Meyle E. Prior B. Wagner C. Hänsch G. M. (2011). Host defence against *Staphylococcus aureus* biofilms by polymorphonuclear neutrophils: oxygen radical production but not phagocytosis depends on opsonisation with immunoglobulin G. Immunobiology 216, 351–357. 10.1016/j.imbio.2010.07.009, PMID: 20850891

[ref105] Sugimoto S. Sato F. Miyakawa R. Chiba A. Onodera S. Hori S.. (2018). Broad impact of extracellular DNA on biofilm formation by clinically isolated methicillin-resistant and -sensitive strains of *Staphylococcus aureus*. Sci. Rep. 8:2254. 10.1038/s41598-018-20485-z, PMID: 29396526 PMC5797107

[ref106] Tebartz C. Horst S. A. Sparwasser T. Huehn J. Beineke A. Peters G.. (2015). A major role for myeloid-derived suppressor cells and a minor role for regulatory T cells in immunosuppression during *Staphylococcus aureus* infection. J. Immunol. 194, 1100–1111. 10.4049/jimmunol.1400196, PMID: 25548227

[ref553] Tecchio C. Micheletti A. Cassatella M. A. (2014). Neutrophil-derived cytokines: facts beyond expression. Front. Immunol. 5:508. 10.3389/fimmu.2014.00508, PMID: 25374568 PMC4204637

[ref107] ten Harkel B. Schoenmaker T. Picavet D. I. Davison N. L. de Vries T. J. Everts V. (2015). The foreign body giant cell cannot resorb bone, but dissolves hydroxyapatite like osteoclasts. PLoS One 10:e0139564. 10.1371/journal.pone.0139564, PMID: 26426806 PMC4591016

[ref108] Thammavongsa V. Kim H. K. Missiakas D. Schneewind O. (2015). Staphylococcal manipulation of host immune responses. Nat. Rev. Microbiol. 13, 529–543. 10.1038/nrmicro3521, PMID: 26272408 PMC4625792

[ref109] Thurlow L. R. Hanke M. L. Fritz T. Angle A. Aldrich A. Williams S. H.. (2011). *Staphylococcus aureus* biofilms prevent macrophage phagocytosis and attenuate inflammation *in vivo*. J. Immunol. 186, 6585–6596. 10.4049/jimmunol.1002794, PMID: 21525381 PMC3110737

[ref110] Triffault-Fillit C. Ferry T. Laurent F. Pradat P. Dupieux C. Conrad A.. (2019). Microbiologic epidemiology depending on time to occurrence of prosthetic joint infection: a prospective cohort study. Clin. Microbiol. Infect. 25, 353–358. 10.1016/j.cmi.2018.04.035, PMID: 29803842

[ref111] Trindade R. Albrektsson T. Tengvall P. Wennerberg A. (2016). Foreign body reaction to biomaterials: on mechanisms for buildup and breakdown of osseointegration. Clin. Implant. Dent. Relat. Res. 18, 192–203. 10.1111/cid.12274, PMID: 25257971

[ref112] Trouillet-Assant S. Gallet M. Nauroy P. Rasigade J.-P. Flammier S. Parroche P.. (2015). Dual impact of live *Staphylococcus aureus* on the osteoclast lineage, leading to increased bone resorption. J. Infect. Dis. 211, 571–581. 10.1093/infdis/jiu386, PMID: 25006047

[ref113] Valour F. Trouillet-Assant S. Rasigade J.-P. Lustig S. Chanard E. Meugnier H.. (2013). *Staphylococcus epidermidis* in orthopedic device infections: the role of bacterial internalization in human osteoblasts and biofilm formation. PLoS One 8:e67240. 10.1371/journal.pone.0067240, PMID: 23840636 PMC3696042

[ref114] Veglia F. Perego M. Gabrilovich D. (2018). Myeloid-derived suppressor cells coming of age. Nat. Immunol. 19, 108–119. 10.1038/s41590-017-0022-x, PMID: 29348500 PMC5854158

[ref115] von Eiff C. Vaudaux P. Kahl B. C. Lew D. Emler S. Schmidt A. (1999). Bloodstream infections caused by small-colony variants of coagulase-negative staphylococci following pacemaker implantation. Clin. Infect. Dis. 29, 932–934. 10.1086/520462 10589914

[ref116] Vuong C. Voyich J. M. Fischer E. R. Braughton K. R. Whitney A. R. DeLeo F. R.. (2004). Polysaccharide intercellular adhesin (PIA) protects *Staphylococcus epidermidis* against major components of the human innate immune system. Cell. Microbiol. 6, 269–275. 10.1046/j.1462-5822.2004.00367.x, PMID: 14764110

[ref117] Wang R. Khan B. A. Cheung G. Y. C. Bach T.-H. L. Jameson-Lee M. Kong K.-F.. (2011). *Staphylococcus epidermidis* surfactant peptides promote biofilm maturation and dissemination of biofilm-associated infection in mice. J. Clin. Invest. 121, 238–248. 10.1172/JCI42520, PMID: 21135501 PMC3007140

[ref118] Wang Y. Liu X. Dou C. Cao Z. Liu C. Dong S.. (2017). Staphylococcal protein A promotes osteoclastogenesis through MAPK signaling during bone infection. J. Cell. Physiol. 232, 2396–2406. 10.1002/jcp.25774, PMID: 28185243 PMC5485048

[ref119] Waters E. M. Rowe S. E. O’Gara J. P. Conlon B. P. (2016). Convergence of *Staphylococcus aureus* persister and biofilm research: can biofilms be defined as communities of adherent persister cells? PLoS Pathog. 12:e1006012. 10.1371/journal.ppat.1006012, PMID: 28033390 PMC5199038

[ref121] Yokota K. Sato K. Miyazaki T. Kitaura H. Kayama H. Miyoshi F.. (2014). Combination of tumor necrosis factor α and interleukin-6 induces mouse osteoclast-like cells with bone resorption activity both *in vitro* and *in vivo*. Arthritis Rheumatol. 66, 121–129. 10.1002/art.38218, PMID: 24431283

[ref122] Zapotoczna M. McCarthy H. Rudkin J. K. O’Gara J. P. O’Neill E. (2015). An essential role for coagulase in *Staphylococcus aureus* biofilm development reveals new therapeutic possibilities for device-related infections. J. Infect. Dis. 212, 1883–1893. 10.1093/infdis/jiv319, PMID: 26044292

[ref123] Zheng Y. Joo H.-S. Nair V. Le K. Y. Otto M. (2018). Do amyloid structures formed by *Staphylococcus aureus* phenol-soluble modulins have a biological function? Int. J. Med. Microbiol. 308, 675–682. 10.1016/j.ijmm.2017.08.010, PMID: 28867522 PMC5832552

